# The WARFUN taboo

**DOI:** 10.1080/07292473.2024.2409538

**Published:** 2024-10-14

**Authors:** Eva Johais

**Affiliations:** Postdoctoral researcher at Chr. Michelsen Institute, Bergen, Norway

**Keywords:** humour, taboo, soldiers, civil–military relations, Germany

## Abstract

The WARFUN taboo rules out the association of war with fun. This article explains why the taboo is particularly entrenched in Germany and demonstrates its effects: the taboo forms part of an anti-militarist political culture and has created soldier models that omit any soldierly qualities that contradict this culture. Oriented towards these models, German soldiers have internalised the taboo as well. However, this contradicts their experience of humour as an integral part of soldier culture. Consequently, soldiers cautiously control what they share with outsiders. Thereby the WARFUN taboo alienates soldiers from the society into which they are supposed to fit.

‘War is no fun’. This rebuke expresses the taboo that rules out any association between war and fun in a nutshell. The rebuke was the last sentence of a personal note in a letter with which the German armed forces (*Bundeswehr*) rejected my application for an official research permit. In the application, I had emphasised that our project title ‘WARFUN’ did not imply that we negated the cruelty and suffering of war and explained how fun served as an analytical entry point into war experience. Despite this effort, the military official in charge stressed that ‘it is evident how inappropriate such play on words is in view of the cruel reality of the war in Ukraine’.^1^

A taboo is a type of social norm that is particularly forceful.^2^ Norms function as mechanisms of social control that guide behaviour according to shared standards of what is appropriate. The ‘regulative effect’ of a taboo consists of preventing something being done, said, or even thought. As taboos follow from how a society defines situations of danger, breaching a taboo must be met by sanctions.^3^ Yet, apart from constraining behaviour, norms also circumscribe proper role models and forms of conduct.^4^ These constitutive effects include unintended consequences that go astray from the desirable function of norms to foster a common social order.

This article examines the taboo that renders it unthinkable to suggest that war offers anything but horror and suffering. Further, it claims that this WARFUN taboo is particularly entrenched and consequential in Germany. While the taboo might apply in other ‘post-heroic’ societies as well, it is far from universal, and its effects depend on a society’s specific relation to war and violence.^5^ This becomes obvious if we consider that the representation of war-making as glorious and honourable, pleasurable and adventurous – the opposite of the WARFUN taboo – pervades state propaganda as well as popular culture in more or less overt forms. The reason is that war functions as a ‘moral arbiter’ for modern states that puts the allegiance of citizens to the ultimate test.^6^ When states apply force in an act of aggression, for self-defence, or as an intervener, it reveals whether citizens are ready to risk their life for a national purpose. Therefore, states invest ‘intense moral work […] to legitimate or condemn particular forms and elements of war, while leaving the war paradigm itself unquestioned’.^7^

In turn, the danger that the WARFUN taboo is supposed to avert is militarisation, understood as the normalisation of ‘military needs and militaristic presumption’.^8^ Claiming that the WARFUN taboo is particularly strong in Germany does not imply that the society is immune to processes of militarisation. Like any social norm, the content and regulative power of the WARFUN taboo is dynamic and does not fully contain all thinking and acting.^9^ Still, it is assumed that the ‘fear of militarisation’ is higher than in other societies because German society has cultivated an awareness of its most devastating consequences.^10^

A challenge for detecting and defining taboos arises from their evasive nature: the unthinkability attached to taboos adds to the tacit quality of ‘normative prohibitions’. Unlike legal proscriptions, social norms are not usually spelled out because they derive from common assumptions and are taken for granted.^11^ Therefore, it is difficult to pin down the taboo and prove its effects.

Accordingly, it has been debated whether the non-use of nuclear weapons by the United States since 1945 is the sign of a taboo or merely conforms to the tradition of confining their use to existential deterrence.^12^ While the issue of using nuclear or chemical weapons belongs in the realm of international norms, the WARFUN taboo qualifies as a cultural taboo, like refraining from the public display of dead bodies.^13^ More precisely, it forms part of the anti-militarist political culture that began to take shape out of the preoccupation with the historical responsibility for waging aggressive wars and committing war crimes after the Second World War.

In the following, this article first explains the salience of the WARFUN taboo in Germany with its political culture and traces its regulative effects. Next, it shows the constitutive effect that the taboo has created soldier models devoid of fun. The final part demonstrates the effect of the WARFUN taboo on soldiers. For this, it refers to soldiers’ reactions when asked about the role of fun and humour in soldier life and illustrates how the WARFUN taboo affects civil–military interactions. The insights on the experience of German soldiers have been gained in the course of research carried out in 2022. This research entailed in-depth interviews and group discussions with thirty-five current or former members of the German armed forces and observations at social events of veteran associations, during visits at Bundeswehr facilities, and at the Bundeswehr Day 2022 in Warendorf. This approach aims at understanding the forms and functions of humour in the everyday life of soldiers.^14^ Finally, the conclusion highlights the unintended side effect that the taboo aggravates the disconnect between soldiers and their social environment.

## Germany’s political culture

Political culture refers to the shared mindset that guides a collective’s orientation towards and action within the political world.^15^ This mindset has a cognitive, an evaluative, and an affective dimension: it encompasses empirical and causal beliefs as well as values, norms, and moral convictions but also feelings of belonging, repugnance, or indifference. In other words, political culture shapes how the collective interprets, judges, and feels about political events and actions. The members of the collective are often unaware of the political culture because they have learned to take the common assumptions for granted through their socialisation into the group.^16^ Furthermore, political culture is a historical product of the collective’s past and present.^17^ The assumptions are reinforced and contested in public struggles over power, legitimacy, and recognition. Actors draw in these struggles on the cultural repertoire that has accumulated over time. Therefore, political cultures are very robust and change gradually rather than radically.^18^ The principled rejection of an association of war with fun – which the personal note cited in the introduction exemplifies – acts out one of the common assumptions that constitute Germany’s political culture. Another outgrowth of this culture is the ‘thought taboo’ against openly considering nuclear options as a part of German security strategy.^19^

It is true that Germany has never been a pacifist state in the sense of having a categorical dismissal of the use of military force.^20^ Instead, characterising its political culture as anti-militarist means that the application of military force is considered an illegitimate means of foreign policy except for self-defence against aggression and as less suited for shaping international relations.^21^ The second element that has characterised German foreign policy is the preference for acting in cooperation with other states in lieu of a self-confident, independent, or leading posture. This reluctant and diplomatic approach has brought Germany a reputation as a ‘civilian power’.^22^

At the heart of Germany’s anti-militarist political culture stands a basic narrative that is defined by a negation of the past.^23^ Prefiguring the identity of the new German state, the parliamentary council which drafted the Basic law for the Federal Republic of Germany (FRG) raised respect for human dignity, pacifism, and anti-nationalism as guiding principles for the state.^24^ In contrast, ‘war’ turned into a synonym for the period of the Nazi regime and, thus, for everything that the founding myth of the FRG negated. Two political maxims encapsulated this rupture with the past: ‘Never again war’ and ‘Never again Auschwitz’.^25^ The maxims translated into a political culture that combined the imperative of military self-restraint and a moral responsibility to prevent genocide and crimes against humanity. While the use of military power was discredited by the atrocities of the Nazi regime, the preference for pursuing international responsibilities through joint action grew out of the attempt to avoid any resemblance to its chauvinist megalomania.

Anti-militarism and multilateralism have remained fairly stable principles of foreign and security policy and can be traced throughout the history of the German armed forces after the Second World War. The underlying maxims have, however, had to be reinterpreted to cope with the new world political situation after 1989 and to allow for a new role for the military. When the Allied powers occupied Germany in May 1945, they disbanded all military troops and dismantled facilities that could be used militarily. But the unfolding Cold War reoriented priorities: The United States supported West Germany’s efforts at rearmament and convinced other Western allies to tolerate the foundation of a new armed force, named the Bundeswehr, in 1955.^26^ The North Atlantic Treaty Organization (NATO) and the Western European Union (WEU) offered a framework for remilitarisation that integrated the Bundeswehr in a system of collective defence and security which tamed its use for pursuing national, let alone expansionist, goals.^27^

In 1990, the disintegration of the Soviet bloc and the unification of the two German states set new terms for national and international politics. First, the Bundeswehr had to integrate soldiers from East Germany’s National People’s Army (NVA). Furthermore, new tasks replaced deterrence as the military’s *raison d’être* and the Bundeswehr started to engage in operations outside NATO territory including mandates for peacekeeping, nation-building, capacity-building of foreign forces, and counter-insurgency.^28^ After small contingents had been sent to Cambodia (1992) and Somalia (1993–4), the discursive shifts by which German politicians and citizens accepted the non-traditional roles of the military happened in the course of the debates on German participation in military interventions in the Yugoslav wars.^29^

In the parliamentary debates on the massacres in Srebrenica in December 1995, the responsibility that derived from the German perpetration of atrocities in the past was reinterpreted as an imperative to intervene and stop atrocities in the present.^30^ Thus, the maxim ‘Never again Auschwitz’ surpassed the principle of military self-restraint. Accordingly, the parliament approved German participation in the NATO-led Implementation Force (IFOR) in Bosnia-Herzegovina. The shift towards a new role for the Bundeswehr as an international intervention force was completed when the parliament decided to support the NATO airstrikes in Kosovo in 1999. In the debates preceding the decision, politicians referred to the German past to dramatise the situation in Kosovo and argue for a moral duty for German politics. The period of National Socialism no longer served as a restraint but as a justification for action. What affected German society’s relation to war more than the missions in Bosnia-Herzegovina (1996–2012)^31^ and Kosovo (since 1999) was the twenty-year-long German operation in Afghanistan (2001–21) because it exceeded the previous operations in terms of scale and nature. The so-called *Karfreitagsgefecht* (‘Good Friday Battle’) on 2 April 2010 marked a turning point in respect of the new quality of the operational reality: it was the first extended experience of combat by German soldiers since the Second World War. But the anti-militarist culture was apparently so ingrained that German politicians thought the public was not ready to confront the reality that German soldiers were being exposed to and applying violence: when German Defence Minister Karl-Theodor zu Guttenberg afterwards suggested for the first time that soldiers had faced a ‘war-like’ situation, even his tentative formulation sparked criticism in the German parliament.^32^ And the hesitancy of the political elite continued, although other public actors openly described the Afghanistan mission as a war.^33^ They toned down the war rhetoric by sticking to the distanced wording of ‘war-like’ and by framing the martial character of the situation as a subjective experience of soldiers rather than a fact.

As the representations of the Afghanistan operation illustrate, it is important to unpack the prevalence and political implications of the anti-militarist culture.^34^ The prevailing views of the populace and of political elites are not necessarily congruent, just as the positions within the spectrum of political parties diverge. In addition, indicators of an anti-militarist attitude among Germans follows no conclusive trend. Public opinion is divided, volatile, and depends on whether the attitude towards the Bundeswehr and its range of tasks, the prerogative of non-military means of foreign policy, or Germany’s global role and responsibility in general, respectively, in response to specific crises, is in question.

Notably, the speech in which German Chancellor Olaf Scholz reacted on 27 February 2022 to the Russian invasion of Ukraine raised doubts about the persistence of the traditional cornerstones of German foreign policy and the prevalence of anti-militarist convictions. He pledged to increase the Bundeswehr’s budget, including a one-time additional sum of 100 billion euros, and reversed the long-held principle of not sending weapons to crisis regions by approving the delivery of weapons to Ukraine, ‘[there]by acknowledging the need for military strength […] Scholz publicly broke a taboo; and he did so to cheering applause, within and outside the Bundestag’.^35^ Thus, it was remarkable not only that Scholz reoriented his foreign policy away from an anti-militarist stance but also that this was met with widespread approval. Still, Scholz did not completely break with Germany’s political culture as he underscored the ‘continuing value of diplomacy and other means of conflict prevention’. Likewise, the reluctance to support military solutions is still ingrained in parts of the German population, as debates and protests concerning weapons deliveries to Ukraine indicate.^36^

Overall, the regulative effect of the WARFUN taboo has not prevented the building and use of military force, but it has curtailed war semantics. The convictions that constitute Germany’s political culture, to which the WARFUN taboo belongs, have been challenged and altered. While this has probably eroded the anti-militarist mindset, it will not vanish overnight.^37^

## German soldier models

In line with this pace of change, the anti-militarist culture has only been cultivated over time. Since the break with the past after the Second World War had not been as radical as the founding myth of the FRG suggests, the effects of the WARFUN taboo on German soldier models have only gradually gained traction. Soldier models refer to conceptions that define demands, skills, and virtues of soldiers. Conceptions include the formal provisions as well as public representations that soldiers confront as frames of reference. The assumption is that soldier models guide soldiers’ conduct to a certain extent because soldiers compare their own experience and behaviour to the available frames of reference.

The claim on the taboo’s constitutive effect on soldier models requires two clarifications: the absence of funny or comic attributes is not limited to German soldier models, and the WARFUN taboo is not the sole reason for this absence. Another reason is that soldier models are supposed to mark the distinction from civilians and design the soldier as righteous and morally superior:

The soldier comes to embody the heroic and virtuous while the civilian becomes seen as a moral bystander, morally inert and even deficient. A set of values have come to be called military values (e.g. discipline, loyalty, honor, integrity, courage, and selflessness) and a set of (dis)values have come to be associated with civilian life (e.g. consumerism, individualism, selfishness, and moral permissiveness).^38^

Furthermore, there are tactical reasons why the military seeks to keep any pictures of soldiers relaxing and enjoying fun activities from the public. It must preserve the image of the tough and professional soldier who can assert her/himself in the operational environment, not least to deter the enemy.^39^ Hence, the discrepancy between the public representation of soldiers and the internal military cultures is neither new nor surprising. Madigan finds that ‘humour was a central, almost defining component of troop culture’ among British soldiers during the First World War.^40^ Civilian war commentaries, however, portrayed the British soldier as a morally superior and courageous hero who displayed a ‘willingness to die’.^41^ Thus, the public discourse and soldiers’ experience created two diametrically opposed versions of heroism:^42^ British soldiers identified with a tragicomical hero figure whose courage consisted of ‘resilient willingness to take whatever the war dished out’ instead of the martial hero ready to sacrifice his life for the greater cause that permeated public representations. The specific constitutive effect of the WARFUN taboo in the German context is that it not only reinforces the omission of the human quality of humour but it has also created soldier models that are compatible with an anti-militarist political culture.

At its core, the debate about the official model for Bundeswehr soldiers has revolved around the question of the exceptionalism of the military profession.^43^ The ‘reformists’ held the view that soldiering was an occupation like any other. In contrast, the ‘traditionalists’ believed that the military stood out by its distinct ethos and status. Accordingly, they claimed an assigned area of responsibility that required special virtues such as courage, discipline, sense of duty, and comradeship. While reformists aim for the civilisation of the military profession, traditionalists advocate for a concentration on core competencies.

The Law on the Status of Soldiers that was adopted in 1956 abolished privileges for soldiers and put them on the same level with all other citizens.^44^ While some traditional soldierly virtues such as obedience and comradeship were preserved, heroic attributes such as honour, courage, and the readiness to fight and die disappeared. From then on, soldiers were subject to the system of values enshrined by the FRG’s Basic Law. The cultural change within the armed forces took time though because the government refrained from a resolute replacement of personnel.^45^ Correspondingly, the tradition decrees that formulated the guiding principles for German soldiers only incrementally moved away from their predecessors.^46^ The first decree in 1965 emphasised the importance of distinctively soldierly competencies and considered the determinacy to fight as one among them. While the decree in 1982 still mentioned the readiness to fight, it evoked the Bundeswehr’s own tradition and distanced it more clearly from the Wehrmacht, the armed force that had served the Nazi regime alongside its SS militias. The most recent decree in 2018 stipulated that the tradition of the Bundeswehr first and foremost derived from its own history since 1955.^47^ Individual German soldiers who served the state prior to 1945 could only be taken as a role model if they had acted in accordance with the values of the FRG’s Basic Law. The current tradition policy hereby primarily refers to those officers who resisted the Nazi regime.

The soldier model that conceptualises the Bundeswehr’s own distinct tradition is the ‘citizen in uniform’. In 2008, it was adopted as the official model in the Bundeswehr’s inner leadership philosophy.^48^ With this, the ‘reformists’ finally asserted themselves, and the WARFUN taboo has demonstrated its constitutive effect. The model of the ‘citizen in uniform’ envisions the soldier as a member of a democratic society who is politically and historically educated and hence takes responsibility for human dignity, freedom, and the rule of law. The civil soldier is stripped of any distinct soldierly qualities, especially any martial attributes. Likewise, the model implies that soldiers do not cultivate an internal military culture – including a special sense of humour – that counteracts their society’s dominant mindset. Yet, as anti-militarist convictions have become a stable, constitutive element of Germany’s political culture, they also extend to the citizens in uniform. Thereby, the model has contributed to the spread of anti-militarism within the armed forces and the concomitant incorporation of the WARFUN taboo by soldiers. The next section will discuss in more detail the unintended side effect that soldiers are, therefore, cautious not to leak any signs of a humour culture that deviates from society’s norms of appropriateness.

Apart from this official model, soldiers compare their experience and behaviour to other models with which the public conveys its perspective on soldiers. Nonetheless, some of the preconceptions that soldiers confront contradict the equalising ideal of the citizen in uniform. Tomforde argues that the public framing rather reinforces the civil–military distinction because it consists of an othering process that separates the ‘experts of violence’ from the normalised civil ‘self’.^49^ The exclusion of soldiers and their professional tasks stems from the marked ‘fear of militarisation of civilian spheres’.^50^ As a result, the first frame that soldiers have to face is being dismissed ‘as murderers’.^51^ This one-sided and hostile attitude towards soldiers became diversified as an effect of the increased media coverage of the mission in Afghanistan.^52^ The public could no longer ignore the fact that soldiers applied military violence in the name of the German state, were exposed to mortal dangers, and were involved in combat action. Therefore, soldiers now appeared as individuals with emotions and experiences. Although the perception of German soldiers became more nuanced, the public frames were still limited. Herzog et al. found that German media reports portrayed soldiers serving in Afghanistan primarily as victims suffering from death, physical or mental injury, and harsh conditions.^53^ An alternative image reconciled the new tasks of German soldiers as international interveners with the ‘citizen in uniform’ model.^54^ As German soldiers have been deployed increasingly in missions outside NATO territory, they have been frequently presented as ‘armed social workers’. This model also pervaded media representations of the Afghanistan mission, which depicted soldiers as peaceful development aides who countered the insurgency by strengthening the Afghan state through civil reconstruction measures.^55^ Media reports only hinted at misconduct discreetly but refrained from representing German soldiers as perpetrators of violence.^56^ If the soldier appeared as a warrior at all, then he acted out of self-defence.^57^

The Bundeswehr’s portrayal of the operation on Facebook likewise sought to avoid irritating an anti-militarist public by excluding the violence and suffering that war entails.^58^ The image of a non-threatening situation under control was achieved through three absences: the absence of enemy fighters, the absence of German soldiers’ emotions, and the absence of physical and mental harm and destruction. In this situation, the soldier appears as an ‘unemotional, rational professional’. Regarding the effects of the WARFUN taboo, the lack of emotional expressions is particularly noteworthy: this not only suppresses any association between war-making and fun but also largely reduces soldiers to their professional role and strips them of any human qualities.

The moderate differentiation of soldier models reflects developments in other ‘postmodern’ Western societies that use their military primarily as an intervention force.^59^ The WARFUN taboo rules out representing German soldiers as having the attributes of a warrior hero. Instead, the hegemonic soldier model that the analysis of the Bundeswehr’s recruitment series ‘*Die Rekruten*’ reveals ‘is a German-specific, civil form of military masculinity [… that] is best described as reluctant’. This self-representation performs the citizen in uniform model and ‘ensure[s] the compatibility of the armed forces and of soldiering as a profession with widespread anti-militarism’.^60^

With the end of the ISAF mission in 2014, the public interest in soldiers’ experiences declined because other operations such as the UN mission in Mali are not covered to the same extent in the media.^61^ Attention has since increased in an ‘emerging social group’, namely veterans who have returned from missions abroad.^62^ How society predominantly frames returning soldiers amounts to another process of othering:^63^ returning soldiers are singled out as ‘marginal men’ who suffer mental health problems and fail to reintegrate back into society. Thereby, society pathologises war experience as an individual, psychological problem and protects itself from discussing its relation to violence.^64^ Overall, it seems that the models available for German soldiers are compatible with the anti-militarist culture, which pays off in high levels of trust for the Bundeswehr as an institution.^65^

## The effect of the WARFUN taboo on soldiers

As mentioned earlier, I was reprimanded for our project title when I applied for a research permit, although I had done my best to explain the serious intention behind it. The rebuke demonstrated that members of the Bundeswehr have internalised the WARFUN taboo. The official was determined to rule out any association between fun and war-making as inappropriate. While other interview partners were more well-disposed and did not dismiss the research interest categorically, they were often sceptical or irritated. ‘To be honest, “war” and “fun” are for me two terms that are mutually exclusive’.^66^ When I inquired more specifically about the role of humour in soldier life, they were often pleasantly surprised. One expressed his gratitude that someone – for the first time – had broached this topic with him as a relation between ‘soldiers and humour is usually ruled out per se’. Furthermore, he got to the heart of the gap between soldier models and soldier experience: ‘Soldiers have humour. Soldiers have a very specific humour which is hardly comprehensible for non-soldiers’.^67^

Another soldier qualified this assessment by pointing out that people outside the Bundeswehr used to be familiar with soldier jokes and jargon due to the closer civil–military relationship in previous times.^68^ But he shared the view that soldier humour would be confined to a ‘closed humour circle’ today. Nonetheless, within this closed circle, soldiers entertain a vibrant humour culture.^69^

Acting this out causes problems since the effect of the WARFUN taboo is that it creates a dilemma for soldiers who experience fun or positive emotions in a war context.^70^ An interlocutor illustrated this dilemma by imitating a scene from a war movie: he pretends to fire a machine gun, with his hands clasping an invisible weapon and his arms shaking with the rhythm of the shooting. The scene he imagines comprises ‘soldiers flying in a helicopter shooting on the poor Vietnamese down in the woods with massive machine guns’. He denounces this iconic war scenario as a ‘perverse form of fun’ and asserts, ‘that’s how you don’t want to experience yourself’. Nevertheless, he disclosed various forms of fun and pleasurable experiences during the interview. Among others, he frankly admitted enjoying shooting and considers the fun of this activity as a self-evident, general fact: ‘Shooting by itself is fun!’^71^ Thus, when he frowns on the imagined scene, he is probably distancing himself not from enjoying shooting but from enjoying shooting *on* and potentially killing someone.

In addition to creating cognitive dissonance, the WARFUN taboo affects civil–military relations. Soldiers reflect on the extent to which they should share their humour culture with outsiders to prevent deviating from civil society and potentially disrupting social relations. For one interlocutor, the WARFUN taboo implied a strict and unquestioned prohibition. He explained that it never would have occurred to him to share moments of fun that he experienced during deployment because that would have been ‘absolutely inappropriate’:

There are two blocs: There is the family that has to do it all alone and is worrying about [you]. And you are on the other side doing your job routinely and [*whispering*] occasionally having fun. [*Back to normal voice*] You can’t do that. Because you assume that the problems and stress at home mix up with [*murmuring*] ‘he has fun’. This idea would have never occurred to me.^72^

Another interview partner described three social situations in which he faced the civil–military humour divide. In the first example he disapproved of a comrade who did not observe this separation. The comrade sent a notification of a change in his private address but formulated it like a military order about changing a command post – presumably to be funny. My interview partner though could not laugh at it. On the contrary, he felt embarrassed because his wife caught sight of the ‘abnormal’ message:

And her reaction: what a weirdo! I was about to apologise for that because I found it weird as well. I mean, nowadays, we also live in the normal society. We are citizens in uniform. Therefore, it is strange if somebody adopts that [military language] in his life to such an extent. […] In a civilian context, it is inappropriate. And it is embarrassing if your wife asks: ‘What kind of guys are around you there?’^73^

The same interview partner recounted the culture shock he experienced during basic training in the late 1980s. The humour his instructors used differed materially from what his family’s social milieu cultivated:

‘And if the sky is full of pussies, nothing moves in the rank [*German: Glied*]’.^74^ I heard this saying 35 years ago and it has become engraved in my memory because I was thinking: ‘What is happening here? Where have I landed?’^75^

While the initial shock did not prevent him from quickly adapting to the new social environment, he understood well that he had to keep the new joke culture separate from his civilian life: ‘I did not dare to tell at home what I heard during the week. My mother was already worried that I had started to drink beer within two weeks as a soldier’.

In another civil–military encounter, he opted instead for authenticity. It is a common custom that soldiers give presents to their instructors at the end of their basic training. Yet the official highlight of basic training is the ceremonial swearing-in. The public – recruits’ parents and relatives – are invited to attend the ceremony and are also shown around the military barracks. For my interlocutor’s commander, this raised the question of how to present military life. He was concerned about the presents displayed in the corridors because many were rather obscene. One was particularly offensive: the recruits had handcrafted a grotesque sculpture of an instructor notorious for his extremely hard training. The sculpture depicted him in the pose of taking a soldier from behind and beating him with a whip. My interlocutor opted against removing the presents, arguing:

It is a little coarse, for sure. But this is everyday life. Many of the parents and siblings used to be soldiers as well. We don’t want to deceive them. We are trying to be authentic. Therefore, I would not like to take it away.^76^

Apparently, he favoured upholding a clear separation in situations that involved his own professional and private life and posed a risk of upsetting his personal relations. Instead, he was less cautious than his commander in view of the open house day, which he understood as an opportunity to offer insights into the reality of military life and culture and where he expected visitors to have an interest in and familiarity with soldier culture.

The unintended side effect of the internalisation of the WARFUN taboo is that it contributes to social exclusion when soldiers cautiously control their interactions with non-soldiers and withhold their military culture. The incommunicability of soldiers’ sense of humour is indicative of the broader disconnection between the military profession and German society that many soldiers described. The strongest experience of disconnection was when they were exposed to the ‘murderer frame’:^77^ ‘You are a murderer and we dislike the Bundeswehr anyway’.^78^ The public attitude towards soldiers would have changed with the war in Ukraine, but there were times, ‘when you outed yourself [as soldier], you were lucky not to get a punch’.^79^

As they encounter rejection, soldiers conceal their identity, especially in supposedly anti-militarist milieus. By way of example, two soldiers recounted a visit to a garden party with ‘remaining hippies’: ‘And she [*name of companion*] whispered to me: Don’t let on anything by all means! We won’t get out alive!’^80^

A less dramatic reason for soldiers’ caution is not fear but an expected lack of interest and understanding:

In fact, we never do what we are doing right now [the interview]. We don’t talk to outsiders. Not even with family because they would not understand as well. We stay among us.^81^

Due to the perceived mismatch with civil society, soldiers close off their professional life from outsiders. The WARFUN taboo aggravates this tendency as it draws a red line that soldiers try not to violate.

## Conclusion

This article sets out why the taboo that renders any association between war and fun unthinkable is particularly ingrained in Germany and demonstrates its regulative and constitutive effects. The WARFUN taboo forms part of Germany’s political culture, which is characterised by anti-militarist convictions and a preference for multilateral action. The WARFUN taboo is supposed to prevent militarisation encroaching on civilian spheres. Germans are particularly aware of this danger due to their historical experience: a nation reputed as the ‘land of poets and thinkers’ betrayed civilisation, and all-out militarisation transformed its civil society into one that waged war against internal and external enemies. Yet the WARFUN taboo has not immunised German society against militarisation and turned it into a pacifist nation. Instead, the anti-militarist stance presumes that the use of military force is less legitimate and effective than other means of foreign policy. As an element of this political culture, the WARFUN taboo has had the regulative effect of containing war semantics and has thereby hampered an open discussion of society’s relation to war and violence.

In addition, the taboo has yielded soldier models which omit the possibility that fun forms an integral part of soldier life. The specific effect in the German context is that soldier models are compatible with the anti-militarist political culture: namely, the official model of the Bundeswehr is the ‘citizen in uniform’ who is a well-integrated member of the democratic society. In addition, both self- and other representations refrain from depicting German soldiers as warriors who perpetrate violence.

Most remarkably, the WARFUN taboo has also been internalised by soldiers themselves in the wake of their socialisation into a predominantly anti-militarist political culture. At the same time, through their military socialisation, soldiers adopt an internal humour culture that they deem inappropriate for outsiders. The tensions between civil and military culture go beyond a state of cognitive dissonance that soldiers everywhere grapple with and entail social consequences. With regard to civil–military relations, the WARFUN taboo translates into soldiers’ hesitancy to share their humour culture. Thereby it reinforces the precarious position of German soldiers, who are torn between the ideal of being a well-integrated ‘citizen in uniform’ and the exposure to ‘othering’ reactions and preconceptions in German society. The desirable effect of the WARFUN taboo is to prevent an unfettered militarisation of civil society. The inversion of this logic, however, creates problems, as the experience of German soldiers shows: the civilianisation of the military profession has limits as it cannot outdo the distinctiveness of the military trade – the readiness to fight and die – that an anti-militarist society prefers to ignore. The unintended side effect of the WARFUN taboo is that it further alienates soldiers from the society into which they are meant to fit.

## Footnotes

1 All translations by the author. The details of this correspondence are not given in order to protect the privacy of her interlocutor.

2 Nina Tannenwald, ‘The Nuclear Taboo: The United States and the Normative Basis of Nuclear Non-Use’, *International Organization* 53, no. 3 (1999), 436–7.

3 Franz Steiner, *Taboo* (Harmondsworth: Penguin, 1967), 20–1, 146–7.

4 Tannenwald, 437.

5 Herfried Münkler, *Der Wandel des Krieges: Von der Symmetrie zur Asymmetrie* (Weilerswist: Velbrück Wissenschaft, 2006), 310 ff.

6 Catherine Lutz and Kathleen Millar, ‘War’, in *A Companion to Moral Anthropology,* edited by Didier Fassin (Hoboken: Wiley-Blackwell, 2012), 482.

7 Lutz and Millar, 482.

8 Cynthia Enloe, *Maneuvers: The International Politics of Militarizing Women’s Lives* (Berkeley: University of California Press, 2000), 2–4.

9 Tannenwald, 435.

10 Maren Tomforde, ‘Framing the Other in Times of War and Terror: Explorations of the Military in Germany’, in *Civil–Military Entanglements: Anthropological Perspectives*, edited by Birgitte Refslund Sørensen and Eyal Ben-Ari (Oxford: Berghahn, 2019), 101.

11 Tannenwald, 436.

12 T.V. Paul, ‘Taboo or tradition? The non-use of nuclear weapons in world politics’, *Review of International Studies* 36, no. 4 (2010), 853–63.

13 Jessica Auchter, ‘On Viewing: The Politics of Looking at the Corpse’, *Global Discourse* 7, no. 2 (2017), 223–38.

14 Thereby, the focus of this research differs from analyses of military recruitment campaigns that employ humorous narrative strategies or humorous stylistic elements: Daniel Beck and Alexander Spencer, ‘Just a Bit of Fun: The Camouflaging and Defending Functions of Humour in Recruitment Videos of the British and Swedish Armed Forces’, *Cambridge Review of International Affairs* 34, no. 1 (2020), 65–84; Frank A. Stengel and David Shim, ‘Militarizing Antimilitarism? Exploring the Gendered Representation of Military Service in German Recruitment Videos on Social Media’, *International Feminist Journal of Politics* 24, no. 4 (2022), 608–31.

15 John S. Duffield, *World Power Forsaken: Political Culture, International Institutions, and German Security Policy after Unification* (Stanford, CA: Stanford University Press, 1998), 23.

16 Gunther Hellmann, Wolfgang Wagner, and Rainer Baumann, *Deutsche Außenpolitik*: *Eine Einführung* (Wiesbaden: Springer, 2014), 194.

17 Michael Schwab-Trapp, *Kriegsdiskurse: Die Politische Kultur des Krieges im Wandel 1991–1999* (Wiesbaden: Springer, 2002), 27.

18 Duffield, 23.

19 Tristan Volpe and Ulrich Kühn, ‘Germany’s Nuclear Education: Why a Few Elites Are Testing a Taboo’, *The Washington Quarterly* 40, no. 3 (2017), 7–27.

20 Jakub Eberle, ‘Germany Has Never Been a Pacifist Power’, <https://foreignpolicy.com/2022/04/04/germany-pacifism-military-defense-ukraine-war-scholz/> [accessed 3 October 2023]; Brian C. Rathbun, ‘The Myth of German Pacifism’, *German Politics & Society* 24, no. 2 (2006), 68–81.

21 Hellmann, Wagner, and Baumann, 200–1.

22 Hans W. Maull, ‘Germany and Japan: The New Civilian Powers’, *Foreign Affairs* 69, no. 5 (1990), 91–106.

23 Schwab-Trapp, 366.

24 Larsen Kempf, ‘Der Soldat als Feindbild im Inneren?’, in *Soldatentum: Auf der Suche nach Identität und Berufung der Bundeswehr heute*, edited by Martin Böcker, Larsen Kempf, and Felix Springer (Munich: Olzog, 2013), 186–7.

25 Schwab-Trapp, 364.

26 Sönke Neitzel, *Deutsche Krieger*: *Vom Kaiserreich zur Berliner Republik – Eine Militärgeschichte* (Berlin: Propyläen, 2020), 313.

27 Sabine Collmer, ‘To Go with the Flow? Change and Persistence in Patterns of Civil–Military Relations in Germany Before and After the End of the Cold War’, in *Armed Forces, Soldiers and Civil–Military Relations: Essays in Honor of Jürgen Kuhlmann*, edited by Gerhard Kümmel, Giuseppe Caforio, and Christopher Dandecker (Wiesbaden: VS Verlag für Sozialwissenschaften, 2009), 163–4.

28 Nina Leonhard, ‘Towards a New German Military Identity? Change and Continuity of Military Representations of Self and Other(s) in Germany’, *Critical Military Studies* 5, no. 4 (2019), 311–12.

29 Collmer, 168–9; Schwab-Trapp, 368, 374.

30 Schwab-Trapp, 364–5.

31 German troops withdrew from the operation EUFOR Althea in 2012 but were redeployed in July 2022 to counter a potential destabilisation of the Balkan states by Russia.

32 Carsten Pietsch, ‘Zur Motivation Deutscher Soldatinnen Und Soldaten Für Den Afghanistaneinsatz’, in *Der Einsatz der Bundeswehr in Afghanistan: Sozial- und Politikwissenschaftliche Perspektiven*, edited by Anja Seiffert, Phil C. Langer, and Carsten Pietsch (Wiesbaden: VS Verlag für Sozialwissenschaften, 2012), 116–17; David Shim and Frank A. Stengel, ‘Social Media, Gender and the Mediatization of War: Exploring the German Armed Forces’ Visual Representation of the Afghanistan Operation on Facebook’, *Global Discourse* 7, no. 2–3 (2017), 333.

33 Anna Geis, ‘The Ambivalence of (Not) Being in a “War”: The “Civilian Power” Germany and the “Stabilization Operation”‘, in *Concepts at Work: On the Linguistic Infrastructure of World Politics*, edited by Piki Ish-Shalom (Ann Arbor: University of Michigan Press, 2021), 78–79.

34 Frank A. Stengel, ‘Bundeswehr und deutsche Gesellschaft: Die Berliner Republik zwischen Militarisierung und Normalisierung’, in *Deutschlands Verteidigungspolitik. Nationale Sicherheit nach der Zeitenwende*, edited by Malte Riemann and Georg Löfflmann (Stuttgart: Kohlhammer, 2023), 139–53.

35 Bernhard Blumenau, ‘Breaking with Convention? *Zeitenwende* and the Traditional Pillars of German Foreign Policy’, *International Affairs* 98, no. 6 (2022), 1911–12.

36 Kate Connolly, ‘Thousands Protest in Berlin against Giving Weapons to Ukraine’, *The Guardian*, 25 February 2023, <https://www.theguardian.com/world/2023/feb/25/thousands-protest-in-berlin-against-giving-weapons-to-ukraine#:∼:text=Thousands%20of%20people%20have%20taken,negotiations%20with%20Vladimir%20Putin%20instead> [accessed 26 October 2023].

37 Hellmann, Wagner, and Baumann, 209.

38 Lutz and Millar, 488.

39 Interview with former German soldier, 8 June 2022.

40 Edward Madigan, ‘“Sticking to a Hateful Task”: Resilience, Humour, and British Understandings of Combatant Courage, 1914–1918’, *War in History* 20, no. 1 (2013), 94.

41 Madigan, 79.

42 Madigan, 91, 95.

43 Nina Leonhard, ‘Über Den (Wesens)Kern des Soldatseins: Professionssoziologische Überlegungen zur gegenwärtigen Debatte um soldatische Berufs- und Selbstbilder im Bereich der Bundeswehr’, in *Professionskulturen – Charakteristika unterschiedlicher professioneller Praxen*, edited by Silke Müller-Hermann et al. (Wiesbaden: Springer, 2018), 14–17.

44 Neitzel, 315.

45 Neitzel, 319.

46 Neitzel, 506, 509–10.

47 Neitzel, 748.

48 Zentrale Dienstvorschrift: Innere Führung. Selbstverständnis und Führungskultur, A-2600/1, Federal Ministry of Defense (28 January 2008).

49 Tomforde, ‘Framing the Other in Times of War and Terror’, 105.

50 Ibid., 101.

51 Ibid., 108–9.

52 Ibid., 102–3, 110–11.

53 Laura M. Herzog et al., ‘Von friedlichen Aufbauhelfern und professionellen Kämpfern: Die Darstellung der deutschen SoldatInnen im Heimatdiskurs’, in *Heimatdiskurs: Wie die Auslandseinsätze der Bundeswehr Deutschland verändern*, edited by Michael Daxner and Hannah Neumann (Bielefeld: Transcript, 2012), 137–66.

54 Neitzel, 582.

55 Herzog et al., 154–5.

56 Ibid., 153–4.

57 Ibid., 155–9.

58 Shim and Stengel, 338–41.

59 Geis, 75.

60 Stengel and Shim, 618, 620–2.

61 Tomforde, ‘Framing the Other in Times of War and Terror’, 110–11.

62 Michael Daxner, Marion Näser-Lather, and Silvia-Lucretia Nicola, eds, *Conflict Veterans*: *Discourses and Living Contexts of an Emerging Social Group* (Newcastle upon Tyne: Cambridge Scholars Publishing, 2018).

63 Tomforde, ‘Framing the Other in Times of War and Terror’, 112–13.

64 Maren Tomforde, ‘“Good Shot”: Gewalterfahrungen von Bundeswehrsoldaten im Auslandseinsatz’, in *Militär Und Gewalt: Sozialwissenschaftliche und ethische Perspektiven*, edited by Nina Leonhard and Jürgen Franke (Berlin: Duncker & Humblot, 2015), 240–1.

65 Meike Wanner, *Das Ansehen der Bundeswehr*: *Persönliche Einstellung versus Meinungsklimawahrnehmung* (Baden-Baden: Nomos, 2019).

66 Interview with former German soldier, 8 June 2022.

67 Interview with former German soldier, 5 April 2022.

68 Interview with German soldier, 17 October 2022.

69 With this finding, my research supports other military studies that acknowledge humour as an integral part of soldier life: Eyal Ben-Ari and Liora Sion, ‘“Hungry, Weary and Horny”: Joking and Jesting among Israel’s Combat Reserves’, *Israel Affairs* 11, no. 4 (2005), 655–71; Thea Aspestrand Bjerke and Nina Rones, ‘The Fine Line between Funny and Offensive Humour in a Total Institution: An Ethnographic Study of Joking Relationships among Army Soldiers’, *Res militaris* 7, no. 2 (2017), 1–23; Richard Godfrey, ‘Soldiering On: Exploring the Role of Humour as a Disciplinary Technology in the Military’, *Organization* 23, no. 2 (2016), 164–83; John Hockey, *Squaddies: Portrait of a Subculture* (Liverpool: Liverpool University Press, 2006 [1986]), 56–7, 72, 136–8; Beate Sløk-Andersen, ‘The Butt of the Joke? Laughter and Potency in the Becoming of Good Soldiers’, *Cultural Analysis* 17, no. 1 (2019), 25–56.

70 Humour culture is understood as one dimension of joyful experiences but is distinct from the thrill and euphoria of combat, the adventure of tours abroad, the delight of homoerotic rituals, or the potential of self-realisation in war. On this: Synne L. Dyvik, ‘“Valhalla Rising”: Gender, Embodiment and Experience in Military Memoirs’, *Security Dialogue* 47, no. 2 (2016), 133–50, <https://doi.org/10.1177/0967010615615730> [accessed 5 March 2024]; Marion Näser-Lather, ‘Experiencing War: The Reconfiguration of the Senses among German Soldiers Deployed to Afghanistan’, *Critical Military Studies* 4, no. 3 (2018), 227–43; Victoria Basham, *War, Identity and the Liberal State: Everyday Experiences of the Geopolitical in the Armed Forces* (London: Routledge, 2013); Thomas Randrup Pedersen, ‘Get Real: Chasing Danish Warrior Dreams in the Afghan “Sandbox”‘, *Critical Military Studies* 3, no. 1 (2017), 7–26.

71 Interview with former German soldier, 18 February 2022.

72 Interview with former German soldier, 8 June 2022.

73 Interview with German soldier, 20 October 2022.

74 ‘*Glied*’ both means being a member or part of a bigger whole and stands for penis. Thus, the instructor combines the military order to fall in line with a sexual joke.

75 Interview with German soldier, 20 October 2022.

76 Interview with German soldier, 20 October 2022.

77 Tomforde, ‘Framing the Other in Times of War and Terror’, 108–9.

78 Interview with former German soldier, 5 April 2022.

79 Interview with two former German soldiers, 22 July 2022.

80 Interview with two former German soldiers, 22 July 2022.

81 Interview with two former German soldiers, 22 July 2022.

